# Acute spontaneous suppurative thyroiditis caused by *Eikenella corrodens* presented with thyrotoxicosis

**DOI:** 10.31744/einstein_journal/2020RC5273

**Published:** 2020-03-18

**Authors:** Pınar Akhanlı, Ömer Bayır, Seyit Murat Bayram, Sema Hepşen, Madamin Badirshaev, Erman Çakal, Güleser Saylam, Mehmet Hakan Korkmaz

**Affiliations:** 1 University of Health Sciences Diskapi Yildirim Beyazit Training and Research Hospital Ankara Turkey University of Health Sciences, Diskapi Yildirim Beyazit Training and Research Hospital, Ankara, Turkey.; 2 Ankara Yildirim Beyazit University Faculty of Medicine Ankara Turkey Ankara Yildirim Beyazit University, Faculty of Medicine, Ankara, Turkey.

**Keywords:** Thyroiditis, Thyrotoxicosis, Eikenella corrodens, Neck

## Abstract

Acute suppurative thyroiditis is a very rare and life-threatening endocrine emergency. Thyrotoxicosis is a rare condition accompanying acute suppurative thyroiditis. While the majority of the cases in the literature are caused by different reasons, spontaneous development is very rare. We present a patient with acute suppurative thyroiditis who presented to our clinic with thyrotoxic findings, and we compared the case to the literature. A 31-year-old male patient was admitted to our clinic with a complaint of progressive neck pain, swelling and redness on midline neck, fever, and palpitations. On physical examination, swelling, redness and tenderness were detected on the neck region that was consistent with the thyroid location. He presented with tremor on the hands, tachycardia and agitation. Thyroid function tests were compatible with thyrotoxicosis, but there were findings supporting the presence of infection in biochemistry tests. On his radiological evaluations, a heterogeneous lesion divided with small septs was observed, with consolidation areas in the left thyroid lobe. In fine needle aspiration biopsy, 2mL of purulent fluid could be aspirated due to the presence of small, separated consolidation areas. He initiated on antibiotic therapy, propranolol, steroid and symptomatic treatment. *Eikenella corrodens* was detected on the culture antibiogram. Antibiotic therapy was continued for 14 days due to less symptoms and better biochemical values. After treatment, the patient had normal thyroid function, had relief of fever and redness of the neck, and was followed-up. It should be kept in mind that acute suppurative thyroiditis may develop spontaneously with the findings of thyrotoxicosis, with no risk factors.

## INTRODUCTION

Acute suppurative thyroiditis (AST) is an extremely rare and life-threatening endocrine emergency, with an incidence of 0.1% to 0.7% in all thyroid diseases.^(1)^ Thyroid hormone levels are generally normal in AST, but thyrotoxicosis is a rare condition.^(2)^Generally, AST develops secondary to systemic diseases, immunodeficiency conditions or traumatic events, such as fine needle aspiration (FNAB), whereas spontaneous AST is quite rare.^(2)^ In this case report, we aimed to discuss a patient who presented with thyrotoxic symptoms to our clinic, and was diagnosed with spontaneous AST.

## CASE REPORT

**Figure 1:**
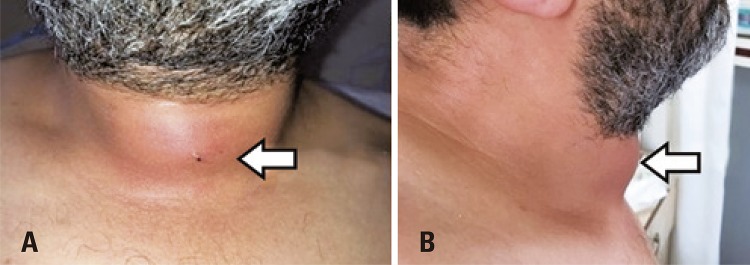
Appearance of the patient’s neck upon admission. (A) Anterior view of the patient’s neck; and (B) Lateral view of the patient’s neck. White arrow: redness, painful swelling of the thyroid

A 31-year-old male patient without any history of chronic diseases, thyroid pathologies or previous neck surgery, was admitted to the ear-nose-throat outpatient clinic with the complaints of neck pain, dysphagia, swelling and redness on midline neck region, fever, and palpitations that started 10 days ago. He had tremor on hands and was generally agitated. The patient had no history of neck trauma and his medical history and family history were uneventful. Blood pressure was 120×70mmHg, axillary temperature 38.5°C, pulse rate 120/minute, respiratory rate 16/minute, and oxygen saturation was 99%. Otolaryngology examination revealed swelling, redness, and tenderness of approximately 7cm, especially in the area corresponding to the thyroid region (Figure 1). In his systemic examination, breath sounds were normal, tachycardia was present, but no pathological sounds or murmurs were detected. In the endoscopic examination, the patient’s nasopharynx, oropharynx, and larynx examinations were normal and there was no fistula in the pyriform sinuses. The other otolaryngology examinations were normal. The patient presented with tremor on the hands and was agitated. Sinus tachycardia was present in the electrocardiogram. In laboratory examinations white blood cell count was 13,200 (75% neutrophil); hemoglobin 15.2g/dL; erythrocyte sedimentation rate (ESR) 54mm/hour; C-reactive protein (CRP) 152mg/L; and liver function tests were minimally elevated. With the suspicion of thyroid diseases, thyroid function tests were analyzed and thyroid-stimulating hormone (TSH) was 0.01µIU/mL (0.38µIU/mL to 5.33µIU/mL), free T4 was 4.9ng/dL (0.58ng/dL to 1.6ng/dL), free T3 was 13.27pg/mL (2.66pg/mL to 4.37pg/mL). These tests showed the patient had thyrotoxicosis.

**Figure 2:**
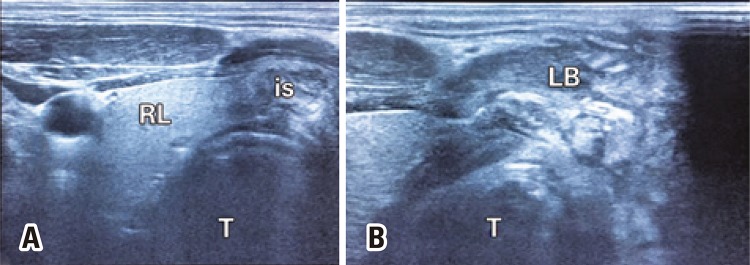
Neck ultrasound of the patient. (A) Axial ultrasound image of thyroid right lobe and isthmus of the patient. The right lobe appears to be normal (B) Axial ultrasound image of the left lobe of the patient. A heterogeneous lesion composed of septated cystic component, intact thyroid parenchyma is not present in the left lobe. Compartments in cystic echogenicity are observed in the left anterior region of the neck, on skin, subcutaneous tissue, and thyroid lobes RL: right lobe; is: isthmus; T: trachea; LB: left lobe.

Anti-thyroglobuline and anti-thyroperoxidase antibodies were negative. Immunoglobulin values were normal. Neck and thyroid ultrasound showed a heterogeneous lesion of 33×34×66mm in left thyroid lobe, formed by septated cystic components, and no robust thyroid parenchyma was seen in the left lobe. In the left anterior neck region, there were compartments with cystic echogenicity on the skin, subcutaneous tissue and thyroid region, while the right thyroid gland parenchyma was normal (Figure 2). The patient was hospitalized.

**Figure 3:**
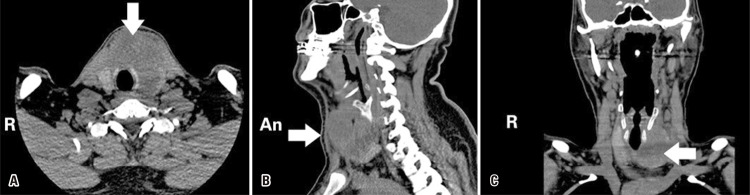
Axial (A), sagittal (B) and coronal (C) non-contrast computed tomography images of the patient. There is a heterogeneous, hypodense lesion located on the left lobe through the isthmus level, and the necrotic-fluid density areas extending from the midline to the right lobe (white arrow) An: anterior; R: right side.

Neck computed tomography with no contrast enhancement revealed a heterogeneous, hypodense lesion, containing some areas of necrotic-liquid density, 65×60×65mm in size, located on the left lobe, along the isthmus level and extending from the midline to the right lobe (Figure 3). Due to the presence of multiple small areas with several septations, only 2mL of purulent fluid could be aspirated in FNAB, and the material was sent for culture antibiogram.

Empirically, treatment was initiated with ampicillin/sulbactam (1,500mg, four times a day, intravenous; Sulcid, İbrahim Etem-Menarini, Istanbul, Turkey) and ornidazole (500mg, twice a day, intravenous; Orniject, Tüm Ekip İlaç, Istanbul, Turkey) for infection. Propranolol hydrochloride (20mg, twice a day, PO; Dideral, Sanofi, Istanbul, Turkey) was started for patient’s agitation, tremor and palpitations. Methylprednisolone (40mg a day, intravenous; Prednol-L, Mustafa Nevzat Pharmaceuticals, Istanbul, Turkey) was started for inflammation of thyroid gland and dexketoprofen trometamol (100mg a day, intravenous; Arveles, İbrahim Etem – Menarini Group, Istanbul, Turkey) for pain and inflammation. Methimazole (5mg, three times a day, PO; Thyromazol, Abdi İbrahim İlaç, Istanbul, Turkey) was prescribed for thyrotoxicosis, as an antithyroid drug.

In culture antibiogram showed *Eikenella corrodens*, susceptible to ampicillin. Ornidazole was discontinued and the treatment was continued with ampicillin/sulbactam. On the 5^th^ day of the treatment, TSH was 0.003µIU/mL, freeT4 3.61ng/dL, freeT3 4.32pg/mL, ESR 35mm/hour, and CRP was 8mg/L. Ampicillin-sulbactam treatment was continued for 14 days, then TSH was 0.01µIU/mL, freeT4 1.14ng/dL, freeT3 2.90pg/mL, ESR 8mm/hour, and CRP was 6mg/L. These findings showed that the patient’s infection was regressing and thyrotoxicosis was getting better.

The patient was discharged 17 days after hospitalization, since he had a complete regression of agitation, tremor, palpitations and painful redness on the neck. Thyroid function tests, ESR and CRP levels were normal at the first month after treatment. The patient was followed as euthyroid in the first year after treatment.

## DISCUSSION

Because of the high vascularization and lymphatic drainage, high iodine concentration and encapsulation, the thyroid gland is resistant to infections and acute thyroid gland infections are extremely rare.^(2)^Acute suppurative thyroiditis may develop due to direct extension of adjacent regions, retropharyngeal abscess, penetrating traumas, thyroid FNAB, intravenous drug usage, rupture or perforation of esophagus, or secondary infections due to immunosuppressive diseases.^(1,2)^Pyriform sinus fistula is a rare condition which is resulting from failure of intrauterine obliteration of third and fourth branchial arch embriologically. The tract of this sinus courses from the pyriform sinus to the thyroid gland, into the perithyroid tissue. The opening of the pyriform sinus fistula can cause AST, especially in the left thyroid lobe.^(3-5)^ The spontaneous development of AST is very rare. In our case, pyriform sinus examination was normal, no brachial cyst was observed. Additionally, the patient had no risk factors for developing AST.

Generally, patients who had AST present with neck swelling, redness of the neck, pain, dysphagia and fever.^(2)^ Differential diagnosis should be made in these patients, especially with subacute thyroiditis and thyroid malignancies, as well as thyroid cyst rupture, sternocleidomastoid abscess and deep vein thrombosis. During the examination, the patient should be monitored quickly and the vital signs should be closely monitored.^(1,2)^Our patient had no previous history of thyroid malignancy, thyroid nodules or cyst, deep vein thrombosis.

Elevated white blood cell count, ESR and CRP levels may be seen in the laboratory findings of AST as in our patient. Patients are usually euthyroid, but may rarely develop temporary or permanent hypothyroidism or thyrotoxicosis due to destruction of the gland.^(2)^Permanent hypothyroidism may occur if the widespread destruction of the gland takes place.^(4,6)^ There is a tendency for euthyroidism in bacterial infections. While most of the patients with bacterial infections are euthyroid, hypothyroidism in fungal infections and hyperthyroidism in mycobacterial infections are more frequent.^(1,7)^

*Staphylococcus* and *Streptococcus* are the most common pathogens in AST (about 40%), and *Gram*-negative agents take the second place (25%).^(7,8)^Methicillin-resistant *Staphylococcus aureus*-identified cases are also present.^(9)^ Acute suppurative thyroiditis is also caused by mycobacterial infections.^(7)^*E. corrodens* is a fastidious, facultative anaerobic *Gram*-negative bacillus, which is found in the gastrointestinal and oral flora, and catalase negative, oxidase positive, urease negative, indole negative. These bacteria can survive in aerobic and anaerobic environments, but cause slow-course infections. They are generally susceptible to ampicillin, chloramphenicol, and tetracycline.^(10)^

Empirical antibiotic therapy in AST should be started without waiting for the results of the culture. The treatment may be only antibiotic therapy in AST or antibiotic treatment after surgical drainage in the presence of abscess. There are some reports of treatment of abscess with aspiration drainage instead of surgical drainage.^(11)^ Surgery should be preferred in the presence of fistula or anatomical problems.^(2,3)^Thyroidectomy can be performed in patients who were presented with diffuse infiltrative, multiple abscess and who have progression of the disease despite antibiotic therapy.^(2)^ Of course, when planning surgical treatment, etiologic factors should be considered. Fistula resection or brachial cyst excision may be added to surgery in patients with pyriform sinus fistula.^(3,4)^In our case, FNAB was performed instead of incisional drainage, due to multiple millimetric abscess foci. Symptomatic and antithyroid drugs were given to reduce the effects of thyrotoxicosis. Antibiotherapy was applied for infection and abscess control.

## CONCLUSION

We reported our diagnosis and treatment approach to a case of spontaneous acute suppurative thyroiditis, which is a very rare disease, accompanied by thyrotoxicosis and caused by *Eikenella corrodens*. It should be kept in mind that, acute suppurative thyroiditis may develop spontaneously with findings of thyrotoxicosis, even with no risk factors. Symptoms of acute suppurative thyroiditis accompanied by thyrotoxicosis may be serious and life-threatening. This potentially fatal picture must be quickly diagnosed, and treatment should be initiated immediately.

## References

[1] 1. Al-Dajani N, Wootton SH. Cervical lymphadenitis, suppurative parotitis, thyroiditis, and infected cysts. Infect Dis Clin North Am. 2007;21(2):523-41, viii. Review. 10.1016/j.idc.2007.03.004 17561081

[2] 2. Pearce EN, Farwell AP, Braverman LE. Thyroiditis. N Engl J Med. 2003; 348(26):2646-55. Review. Erratum in: N Engl J Med. 2003;349(6):620. 10.1056/NEJMra021194 12826640

[3] 3. Pereira KD, Losh GG, Oliver D, Poole MD. Management of anomalies of the third and fourth branchial pouches. Int J Pediatr Otorhinolaryngol. 2004; 68(1):43-50. 10.1016/j.ijporl.2003.09.004 14687686

[4] 4. Paes JE, Burman KD, Cohen J, Franklyn J, McHenry CR, Shoham S, et al. Acute bacterial suppurative thyroiditis: a clinical review and expert opinion. Thyroid. 2010;20(3):247-55. Review. 10.1089/thy.2008.0146 20144025

[5] 5. Kondo T. Acute suppurative thyroiditis secondary to pyriform sinus fistula. Lancet Infect Dis. 2019;19(4):447. 10.1016/S1473-3099(18)30657-1 30938300

[6] 6. Türkiye Endokrin ve Metabolizma Hastalıkları Derneği. Tiroid Çalışma Grubu Tiroid Hastalıkları Tanı ve Tedavi Klavuzu 2017. Tiroiditler. Ortadoğu Yayıncılık. 2017; p. 89-103.

[7] 7. Yu EH, Ko WC, Chuang YC, Wu TJ. Suppurative Acinetobacter baumanii thyroiditis with bacteremic pneumonia: case report and review. Clin Infect Dis. 1998;27(5):1286-90. Review. 10.1086/514998 9827283

[8] 8. Yedla N, Pirela D, Manzano A, Tuda C, Lo Presti S. Thyroid Abscess: Challenges in Diagnosis and Management. J Investig Med High Impact Case Rep. 2018;6:2324709618778709. 10.1177/2324709618778709 PMC5971374 29854858

[9] 9. Lethert K, Bowerman J, Pont A, Earle K, Garcia-Kennedy R. Methicillin-resistant Staphylococcus aureus suppurative thyroiditis with thyrotoxicosis. Am J Med. 2006;119(11):e1-2. 10.1016/j.amjmed.2006.03.016 17071148

[10] 10. Lacroix JM, Walker C. Characterization of a beta-lactamase found in Eikenella corrodens. Antimicrob Agents Chemother. 1991;35(5):886-91. 10.1128/aac.35.5.886 PMC245124 1854171

[11] 11. Ilyin A, Zhelonkina N, Severskaya N, Romanko S. Nonsurgical management of thyroid abscess with sonographically guided fine needle aspiration. J Clin Ultrasound. 2007;35(6):333-7. 10.1002/jcu.20288 17471585

