# Supplementary material for: Acute spontaneous suppurative thyroiditis caused by Eikenella corrodens presented with thyrotoxicosis
Source: Einstein (Sao Paulo). 2020 Mar 18;18:eRC5273. doi: 10.31744/einstein_journal/2020RC5273 (PMC7087456; doi:10.31744/einstein_journal/2020RC5273)
Supplement: Translation

# Translation: Tireoidite supurativa espontânea aguda causada por *Eikenella corrodens* acompanhada de tireotoxicose

**Authors:** Pınar Akhanlı, Ömer Bayır, Seyit Murat Bayram, Sema Hepşen, Madamin Badirshaev, Erman Çakal, Güleser Saylam, Mehmet Hakan Korkmaz

**Affiliations:** 1 Turkey University of Health Sciences, Diskapi Yildirim Beyazit Training and Research Hospital, Ankara, Turkey.; 2 Turkey Ankara Yildirim Beyazit University, Faculty of Medicine, Ankara, Turkey.

**Keywords:** Tireoidite, Tireotoxicose, Eikenella corrodens, Pescoço

## Abstract

A tireoidite supurativa aguda é uma emergência endócrina muito rara e com risco de vida. A tireotoxicose é uma doença rara, que acompanha a tireoidite supurativa aguda. A maioria dos casos descritos na literatura tem diferentes causas, mas o desenvolvimento espontâneo é muito raro. Relatamos o caso de um paciente com tireoidite supurativa aguda, que veio a nossa clínica apresentando achados tireotóxicos, e o comparamos com a literatura. Trata-se de paciente do sexo masculino, 31 anos, que foi internado em nossa clínica com queixa de dor progressiva, edema e vermelhidão na linha média do pescoço, febre e palpitações. Ao exame físico, foram observados edema, vermelhidão e sensibilidade à dor na região do pescoço, consistente com a localização da tireoide. Apresentava tremor de mãos, taquicardia e agitação. Embora os exames de função tireoidiana fossem compatíveis com tireotoxicose, houve achados que sustentavam a presença de infecção nos exames bioquímicos. Nas avaliações radiológicas, observou-se lesão heterogênea dividida por pequenos septos, com áreas de consolidação no lobo tireoidiano esquerdo. Na biópsia por aspiração por agulha fina, foi possível aspirar apenas 2mL de líquido purulento, devido à presença de pequenas áreas de consolidação separadas umas das outras. Iniciaram-se antibioticoterapia, administração de propranolol e esteroides, além de tratamento sintomático. *Eikenella corrodens* cresceu na cultura do antibiograma. A antibioticoterapia foi estendida por 14 dias devido à melhora nos sintomas e dos valores bioquímicos. Após o tratamento, o paciente se apresentava eutireoideo, com melhora na febre e na vermelhidão no pescoço, sendo então acompanhado. Deve-se ter em mente que a tireoidite supurativa aguda pode se desenvolver espontaneamente com achados de tireotoxicose, sem nenhum fator de risco.

## INTRODUÇÃO

A tireoidite supurativa aguda (TSA) é uma emergência endócrina extremamente rara e com risco de vida, com incidência de 0,1% a 0,7% entre as doenças da tireoide.^(1)^ Os níveis de hormônios tireoidianos geralmente são normais na TSA, mas a tireotoxicose é uma doença rara.^(2)^Geralmente, a TSA se desenvolve secundariamente a doenças sistêmicas, imunodeficiência ou eventos traumáticos, como punção aspirativa com agulha fina (PAAF), ao passo que a TSA espontânea é bastante rara.^(2)^ Neste relato de caso, propomos discutir sobre um paciente que procurou nossa clínica apresentando sintomas tireotóxicos e foi diagnosticado com TSA espontânea.

## RELATO DE CASO

Paciente do sexo masculino, 31 anos, sem histórico de doenças crônicas, patologias da tireoide ou cirurgia prévia do pescoço, foi admitido no ambulatório de otorrinolaringologia com queixas de dor no pescoço, disfagia, edema e vermelhidão na região da linha média do pescoço, febre e palpitações que começaram 10 dias antes. Apresentava tremor nas mãos e estava agitado. O paciente não tinha história de trauma no pescoço, nem história pregressa ou familiar importante. A pressão arterial era 120×70mmHg, temperatura axilar 38,5°C, pulso de 120/minuto, frequência respiratória de 16/minuto, e saturação de oxigênio de 99%. O exame otorrinolaringológico revelou edema, vermelhidão e sensibilidade em uma extensão de aproximadamente 7cm, principalmente na área correspondente à região tireoidiana (Figura 1). No exame médico geral, a ausculta respiratória era normal, com taquicardia presente, mas sem serem detectados bulhas ou sopros patológicos. No exame endoscópico, nasofaringe, orofaringe e laringe do paciente eram normais, e não havia fístula nos seios piriformes. Os demais exames otorrinolaringológicos foram normais, e o paciente apresentava tremor nas mãos e, em seu estado geral, mostrava-se agitado. O eletrocardiograma mostrava taquicardia sinusal. Nos exames laboratoriais, a contagem de glóbulos brancos era de 13.200 (75% de neutrófilos); hemoglobina 15,2g/dL; velocidade de hemossedimentação (VHS) 54mm/h; proteína C-reativa (PCR) 152mg/L; e os exames de função hepática apresentavam elevação mínima. Havendo suspeita de doenças da tireoide, foram analisados os exames de função tireoidiana, e a dosagem de hormônio tireoestimulante (TSH) foi de 0,01µUI/mL (0,38µUI/mL a 5,33µUI/mL), com T4 livre de 4,9ng/dL (0,58ng/dL a 1,6ng/dL), e T3 livre de 13,27pg/mL (2,66pg/mL a 4,37pg/mL). Esses exames mostraram que o paciente apresentava tireotoxicose.

**Figura 1:** Aparência do pescoço do paciente no momento da admissão. (A) Vista anterior do pescoço do paciente; e (B) Vista lateral do pescoço do paciente. Seta branca: vermelhidão e inchaço doloroso na região da tireoide

Os anticorpos antitireoglobulina e antitireoperoxidase foram negativos. Os valores de imunoglobulina estavam normais. A ultrassonografia do pescoço e da tireoide mostrou lesão heterogênea de 33×34×66mm no lobo esquerdo da tireoide, com componentes císticos septados, não sendo observado parênquima tireoidiano intacto no lobo esquerdo. Na região anterior esquerda do pescoço, havia compartimentos com ecogenicidade cística na pele, tecido subcutâneo e região da tireoide, ao passo que o parênquima da glândula tireoide direita era normal (Figura 2). O paciente foi hospitalizado.

**Figura 2:** Ultrassonografia do pescoço do paciente. (A) Imagem de ultrassonografia axial do lobo direito e istmo da tireoide do paciente. O lobo direito parece normal. (B) Imagem de ultrassonografia axial do lobo esquerdo da tireoide do paciente. Lesão heterogênea formada por componente cístico septado. Não há parênquima tireoidiano intacto no lobo esquerdo. São observados compartimentos de ecogenicidade cística na região anterior esquerda do pescoço, na pele, no tecido subcutâneo e nos lobos da tireoide RL: *rigth lobe*; is: istmo; T: traqueia; LB: lobo esquerdo.

A tomografia computadorizada do pescoço sem contraste revelou lesão heterogênea e hipodensa, contendo algumas áreas com densidade líquido-necrótica, medindo 65×60×65mm, localizadas no lobo esquerdo ao longo do nível do istmo e se estendendo da linha média ao lobo direito (Figura 3). Devido à presença de múltiplas áreas pequenas com diversas septações, apenas 2mL de líquido purulento foram aspirados na PAAF, e o material foi enviado para antibiograma da cultura.

**Figura 3:** Imagem axial (A), sagital (B) e coronal (C) de tomografia computadorizada sem contraste do paciente. Há uma lesão hipodensa e heterogênea localizada no lobo esquerdo, ao longo do nível do istmo e nas áreas de densidade de líquido necrótico, que se estendem da linha média ao lobo direito (seta branca) An: anterior; R: lado direito.

Empiricamente, foi iniciado tratamento com ampicilina/sulbactam (1.500mg, quatro vezes dia, por via intravenosa; Sulcid, İbrahim Etem-Menarini, Istambul, Turkey) e ornidazol (500mg, duas vezes ao dia, por via intravenosa; Orniject, Tüm Ekip İlaç, Istambul, Turkey) para infecção. Foi iniciada a administração de cloridrato de propranolol (20mg, duas vezes ao dia, por via oral; Dideral, Sanofi, Istambul, Turkey) devido à agitação, aos tremores e às palpitações do paciente. Iniciaram-se também metilprednisolona (40mg ao dia, por via intravenosa; Prednol-L, Mustafa Nevzat Pharmaceuticals, Istambul, Turkey) para a inflamação da glândula tireoide, e dexcetoprofeno trometamol (100mg ao dia, por via intravenosa; Arveles, Grupo İbrahim Etem – Menarini, Istambul, Turkey), para dor e inflamação. Metimazol (5mg, três vezes ao dia, por via oral; Thyromazol, Abdi İbrahim İlaç, Istambul, Turkey), para tireotoxicose, foi usado como medicação antitireoidiana.

No antibiograma da cultura, cresceu *Eikenella corrodens*, sensível à ampicilina. Ornidazol foi interrompido, e o tratamento prosseguiu com ampicilina/sulbactam. No quinto dia de tratamento, TSH era 0,003µUI/mL, T4 livre 3,61ng/dL, T3 livre 4,32pg/mL, VHS 35mm/hora e PCR 8mg/L. O tratamento com ampicilina/sulbactam foi continuado por 14 dias, quando, então, TSH era de 0,01µUI/mL, T4 livre 1,14ng/dL, o T3 livre 2,90pg/mL, VHS 8mm/hora e PCR 6mg/L. Esses achados mostraram que a infecção do paciente estava regredindo, e a tireotoxicose estava melhorando.

O paciente recebeu alta 17 dias após a hospitalização, com regressão completa da agitação, dos tremores, das palpitações e da vermelhidão e dor no pescoço. Os exames de função tireoidiana, VHS e PCR foram normais no primeiro mês após o tratamento. O paciente se mostrou eutireoideo durante o acompanhamento, no primeiro ano após o tratamento.

## DISCUSSÃO

Devido à alta vascularização, à drenagem linfática, à alta concentração de iodo e ao encapsulamento, a glândula tireoide é resistente a infecções, sendo extremamente rara a ocorrência de infecções agudas dessa glândula.^(2)^ A TSA pode se desenvolver devido à extensão direta de regiões adjacentes, abscesso retrofaríngeo, traumas penetrantes, PAAF da tireoide, uso de drogas intravenosas, ruptura ou perfuração do esôfago ou infecções secundárias devido a doenças imunossupressoras.^(1,2)^ A fístula do seio piriforme é doença rara, resultante da falha na obliteração intrauterina do terceiro e do quarto arcos branquiais embrionários. O trato desse seio vai do seio piriforme à glândula tireoide, passando para dentro do tecido peritireoidiano. A abertura da fístula do seio piriforme pode causar TSA, principalmente no lobo tireoidiano esquerdo.^(3-5)^O desenvolvimento espontâneo da TSA é muito raro. Em nosso caso, o exame do seio piriforme foi normal, não sendo observado cisto branquial. Além disso, o paciente não apresentava fatores de risco para o desenvolvimento de TSA.

Geralmente, os pacientes que apresentaram TSA têm edema e vermelhidão no pescoço, dor, disfagia e febre.^(2)^ Nesses pacientes, o diagnóstico diferencial deve ser feito principalmente com tireoidite subaguda e malignidade da tireoide, além de ruptura de cisto da tireoide, abscesso no músculo esternocleidomastoideo e trombose venosa profunda. Durante o exame, o paciente deve ser rapidamente monitorado, e os sinais vitais devem ser monitorados de perto.^(1,2)^Nosso paciente não tinha história prévia de malignidade da tireoide, nódulos ou cistos da tireoide, nem trombose venosa profunda.

Podem ser observados contagem elevada de glóbulos brancos e níveis elevados de VHS e de PCR nos achados laboratoriais da TSA, como ocorreu em nosso paciente. Os pacientes geralmente são eutireoideos, mas raramente se desenvolve hipotireoidismo temporário ou permanente ou tireotoxicose devido à destruição da glândula.^(2)^ Pode ocorrer hipotireoidismo permanente, se houver destruição generalizada da glândula.^(4,6)^ Há uma tendência para o eutireoidismo nas infecções bacterianas. Embora os pacientes com infecções bacterianas, em sua maioria, sejam eutireoideos, é mais frequente haver hipotireoidismo, nas infecções fúngicas, e hipertireoidismo, nas infecções micobacterianas.^(1,7)^

*Staphylococcus* e *Streptococcus* são os patógenos mais comuns na TSA (aproximadamente 40%), e os *Gram*-negativos ocupam o segundo lugar (25%).^(7,8)^ Há casos identificados de *Staphylococcus aureus* resistentes à meticilina.^(9)^ A TSA também é causada por infecções micobacterianas.^(7)^*E. corrodens* é um bacilo *Gram*-negativo anaeróbico facultativo e exigente, encontrado nas floras gastrintestinal e oral, sendo também catalase negativo, oxidase positiva, urease negativo e indol negativo. Essas bactérias podem sobreviver em ambientes aeróbicos e anaeróbicos, mas causam infecções de evolução lenta. Geralmente são sensíveis a ampicilina, cloranfenicol e tetraciclina.^(10)^

A antibioticoterapia empírica na TSA deve ser iniciada sem aguardar os resultados da cultura. O tratamento da TSA pode consistir apenas em antibioticoterapia, ou na administração de antibióticos após drenagem cirúrgica, na presença de abscesso. Existem relatos de tratamento do abscesso com drenagem aspirativa em vez de drenagem cirúrgica.^(11)^ Deve-se dar preferência à cirurgia na presença de fístula ou problemas anatômicos.^(2,3)^A tireoidectomia pode ser realizada em pacientes que apresentarem infiltrado difuso, abscesso múltiplo e progressão da doença, apesar da antibioticoterapia.^(2)^Evidentemente, ao planejar o tratamento cirúrgico, devem ser levados em consideração os fatores etiológicos. A ressecção da fístula ou a excisão do cisto braquial podem ser adicionados à cirurgia em pacientes com fístula do seio piriforme.^(3,4)^Em nosso caso, foi realizada PAAF em vez da drenagem incisional, devido à existência de múltiplos focos de abscesso milimétricos. Foram administradas medicações sintomáticas e antitireoidianas para reduzir os efeitos da tireotoxicose. Utilizou-se antibioterapia para controle da infecção e do abscesso.

## CONCLUSÃO

Relatamos nossa abordagem para diagnóstico e tratamento de um caso espontâneo de tireoidite aguda supurativa (uma doença muito rara) acompanhada de tireotoxicose e causada por *Eikenella corrodens*. Deve-se ter em mente que a tireoidite supurativa aguda pode se desenvolver espontaneamente com achados de tireotoxicose, sem que haja fator de risco. Os sintomas da tireoidite supurativa aguda acompanhada de tireotoxicose podem ser graves e representar risco de vida. Esse quadro potencialmente fatal deve ser diagnosticado rapidamente, e o tratamento deve ser iniciado imediatamente.
